# Aberrant Interference of Auditory Negative Words on Attention in Patients with Schizophrenia

**DOI:** 10.1371/journal.pone.0083201

**Published:** 2013-12-23

**Authors:** Norichika Iwashiro, Noriaki Yahata, Yu Kawamuro, Kiyoto Kasai, Hidenori Yamasue

**Affiliations:** 1 Department of Neuropsychiatry, Graduate School of Medicine, The University of Tokyo, Bunkyo-ku, Tokyo, Japan; 2 Global Center of Excellence (COE) Program, The University of Tokyo, Bunkyo-ku, Tokyo, Japan; 3 Takada-Nishishiro Hospital, Jyoetsu-shi, Niigata, Japan; 4 JST, National Bioscience Database Center (NBDC), Chiyoda-ku, Tokyo, Japan; UNLV, United States of America

## Abstract

Previous research suggests that deficits in attention-emotion interaction are implicated in schizophrenia symptoms. Although disruption in auditory processing is crucial in the pathophysiology of schizophrenia, deficits in interaction between emotional processing of auditorily presented language stimuli and auditory attention have not yet been clarified. To address this issue, the current study used a dichotic listening task to examine 22 patients with schizophrenia and 24 age-, sex-, parental socioeconomic background-, handedness-, dexterous ear-, and intelligence quotient-matched healthy controls. The participants completed a word recognition task on the attended side in which a word with emotionally valenced content (negative/positive/neutral) was presented to one ear and a different neutral word was presented to the other ear. Participants selectively attended to either ear. In the control subjects, presentation of negative but not positive word stimuli provoked a significantly prolonged reaction time compared with presentation of neutral word stimuli. This interference effect for negative words existed whether or not subjects directed attention to the negative words. This interference effect was significantly smaller in the patients with schizophrenia than in the healthy controls. Furthermore, the smaller interference effect was significantly correlated with severe positive symptoms and delusional behavior in the patients with schizophrenia. The present findings suggest that aberrant interaction between semantic processing of negative emotional content and auditory attention plays a role in production of positive symptoms in schizophrenia. (224 words)

## Introduction

Disconnection between different elements of brain networks or cognitive domains characterizes the schizophrenia diathesis [1], [2], [3]. For example, deficits in attention-emotion interaction as well as those in each domain have repeatedly been investigated in patients with schizophrenia [4], [5], [6], [7], [8], [9], [10], [11]. Previous studies have shown that presentations of emotionally negative stimuli such as angry face or prosody interfere with attention and lead to slower response in healthy subjects [12], [13], [14], [15]. Patients with schizophrenia show significant deviations in this interference effect of emotionally negative stimuli [4], [7], [9].

Dysfunctional language-related information processing such as aberrant semantic processing plays a role in the pathophysiology of schizophrenia [16], [17], [18], particularly in the formation of positive symptoms such as delusions [19], [20], [21]. Therefore, visually presenting language stimuli has often been utilized to examine the interaction between attention and emotional processing in patients with schizophrenia (reviewed in [22]). However, disruption in auditory information processing is considered crucial in the pathophysiology of schizophrenia, with an emphasis on auditory hallucinations in diagnostic criteria [23], [24] and with various cognitive deficits associated with auditory processing [25], [26], [27]. Taken together, investigating the interaction between emotional processing of auditorily presented language stimuli and attention on auditory perception would bring novel and significant findings in the pathophysiology of psychotic symptoms in schizophrenia.

Previous studies [12], [13] investigated interaction between auditory spatial attention and emotional processing utilizing dichotic listening paradigm and showed prolonged reaction time when angry prosody presented in left ear but only when this side was to-be-attended in healthy subjects. The current dichotic listening study utilized semantically emotional words instead of emotional prosody to approach the pathophysiology of aberrant semantic processing in schizophrenia. Based on the previous prosody studies, we predicted that 1) response time would be longer when negative but not positive words were auditorily presented than when only neutral ones were presented in healthy subjects; 2) subjects with schizophrenia would show abnormality in this interference effect of auditory negative words compared with healthy subjects; and 3) the abnormality in interference effect of auditory negative words would be associated with severity of psychotic symptoms in patients with schizophrenia.

## Materials and Methods

### Ethics statement

The ethics committee of Takada-Nishishiro Hospital approved this study. Written informed consent was obtained from all participants after they were given a complete explanation of the study as approved by the ethics committee. For participants below the age of 20 years, we obtained written informed consent from his or her parents as well as from the participants themselves. All participants belong to patient group were determined by their psychiatrist in charge to have the mental and intellectual capacity to give written informed consent prior to participation in the study. If a participant or their parents could not understand these issues during explanation, s/he was not asked to participate in this research.

### Participants

Forty-six Japanese adults participated in this study. Of these, 22 in- and out-patients with schizophrenia were recruited from the Takada-Nishishiro Hospital, Japan. Diagnosis was confirmed as schizophrenia with the Structured Clinical Interview for DSM-IV Axis I Disorder Clinical Version [24]. Psychiatric symptoms were evaluated using the Positive and Negative Syndrome Scale (PANSS) [28] just before the current psychological experiment. Delusional behavior scores were calculated from subscales of the PANSS. The calculation algorithm was P1 (delusion) +P5 (grandiosity) +P6 (suspiciousness) +G9 (unusual thought content) [29], [30], [31]. All clinical evaluations were conducted by a psychiatrist (N.I.) fully trained to maintain reliability and consistency in diagnoses and evaluation of symptom severity. Twenty of the 22 patients were categorized as paranoid subtype; the other two were categorized as disorganized subtype. Intelligence quotient (IQ) was evaluated using the Japanese version of the National Adult Reading Test [32], [33], [34]. Based on previous studies, dexterous ear was decided using four question items (Appendix S1) [35], [36], [37].

Twenty-four healthy subjects were employed as controls. The control group was matched to the subjects with schizophrenia in age, sex ratio, parental socioeconomic status (SES) [38], handedness [39], dexterous ear, and estimated premorbid IQ. The controls were screened for neuropsychiatric disorders using the Structured Clinical Interview for DSM-IV Axis I Disorder Non-Patient Edition [23], [40].

Exclusion criteria for both groups were current or past neurological or audiological illness, traumatic brain injury with any known cognitive consequences or loss of consciousness for more than 5 minutes, history of electroconvulsive therapy, and substance abuse or addiction. To screen for participants with potential auditory abnormalities, we tested hearing ability in both ears. The test comprised a 1000 Hz tone at 30 dB and a 4000 Hz tone at 40 dB tone, as measured by audiometer (Minato; http://www.minato-med.co.jp/products/inspect/amc1.html). This test was taken from the Japanese Ordinance on Industrial Safety and Health No. 44 (Ordinance of the Ministry of Health, Labour and Welfare) (e.g. https://www.rouki.or.jp/furukawa/modules/mydownloads/jyouhou/ryoukin.pdf). An additional exclusion criterion for the control group was history of psychiatric disease in self or a family history of Axis I disorder in a first-degree relative. The ethics committee of Takada-Nishishiro Hospital approved this study. After a complete explanation of the study, written informed consent was obtained from every individual (Table 1).

**Table 1:** Subject characteristics and symptom scores.

	Patients with Schizophrenia (n = 22)		Controls (n = 24)		T-test	
	Mean	SD	Mean	SD	T-value	P-value
Age (Range)	31.6 (20–40)	5.2	29.9 (18–39)	5.7	1.1	0.28
Male/Female	11/11		11/13		0.08 (x^2^)	0.78 (x^2^)
SES^a^	3.9	1.0	2.7	0.8	4.7	0.00
Parental SES	3.0	0.8	3.1	0.7	−0.4	0.70
Years of education	13.5	1.8	14.4	1.4	1.72	0.09
IQ (JART25)^b^	96.7	10.0	99.3	8.1	−1.0	0.34
Handedness^c^	89.5	34.5	98.0	4.8	−1.2	0.24
Dexterous ear (right/mixed/left)	18/1/3		16/5/3		2.7 (x^2^)	0.26 (x^2^)
Neuroleptic dose^d^ (mg/day)	709.4	641.8				
Onset of illness (years)	24.3	6.3				
Duration of illness (months)	104.1	80.5				
PANSS^e^ Positive symptoms	17.0	2.7				
PANSS^e^ Negative symptoms	23.5	4.6				
PANSS^e^ General psychopathology	42.5	6.9				
PANSS^e^ Delusional behavior	9.9	1.9				

a Socioeconomic status, assessed using the Hollingshead scale. Higher scores indicate lower statu;

b Estimated from scores on the Japanese Adult Reading Test;

c Assessed using the Edinburgh Inventory. >0 indicates right-handed;

d Based on chlorpromazine equivalents;

e Positive and Negative Syndrome Scale.

### Experimental procedure

We selected three word categories of emotional valence (20 different negative/60 different neutral/20 different positive words) from the Affective Norms for English Words (ANEW; [41]) and translated them to Japanese (Appendix S2). All the words were nouns and consisted of four morae in Japanese (Appendix S3). For validation, 20 healthy adults (10 male/10 female) not participating in the current experiment rated these words in terms of their valence, arousal, and dominance by the same 9-point Likert scale as in the ANEW [41]. Word frequency was quoted from a Japanese database [42], and number of orthographic neighbours was calculated from a Japanese thesaurus [43]. The current experiment used three types of word lists that significantly differed only in emotional valence (negative/neutral/positive) (ANOVA; p<0.001), but were equal in all other language features such as arousal (p = 0.15), dominance (p = 0.72), word frequency (p = 0.25), and number of orthographic neighbours (p = 0.22) (ref. [41], [44]). All the words were transformed to auditory stimuli pronounced by synthesized voice in neutral prosody using VoiceText® (HOYA; http://voicetext.jp/). We then processed each auditory stimulus to equalize the duration of the utterances into 700 milliseconds, which were spoken in a naturalistic way using Sound Forge Pro 10® (Sony Creative Software; http://www.sonycreativesoftware.com/soundforgepro). The amplitude (negative; positive; neutral, mean/SD/range = −77.6/6.3/−90.3 to −67.4; −79.6/7.9/−66.8 to −90.3; −77.4/7.0/−65.2 to −90.3 dB) and pitch (negative; positive; neutral, mean/SD/range = 925.7/640.2/288.5–2679.0; 870.4/538.4/374.2–1925.7; 1035.3/646.8/295.7–2468.4 Hz) did not significantly differ among the negative, positive, and neutral word categories (F[2,89] = 0.69, p = 0.50 and F[2,97] = 0.62, p = 0.54 respectively). In addition, the mean acoustic energies (RMS) of the two words, which were presented simultaneously in a dichotic paradigm, were always matched using Mitsyn® (WLH) and CoolEdit Pro® (Syntrillium Software Corporation), according to previous studies [12], [13], [45], [46].

**Figure 1:**
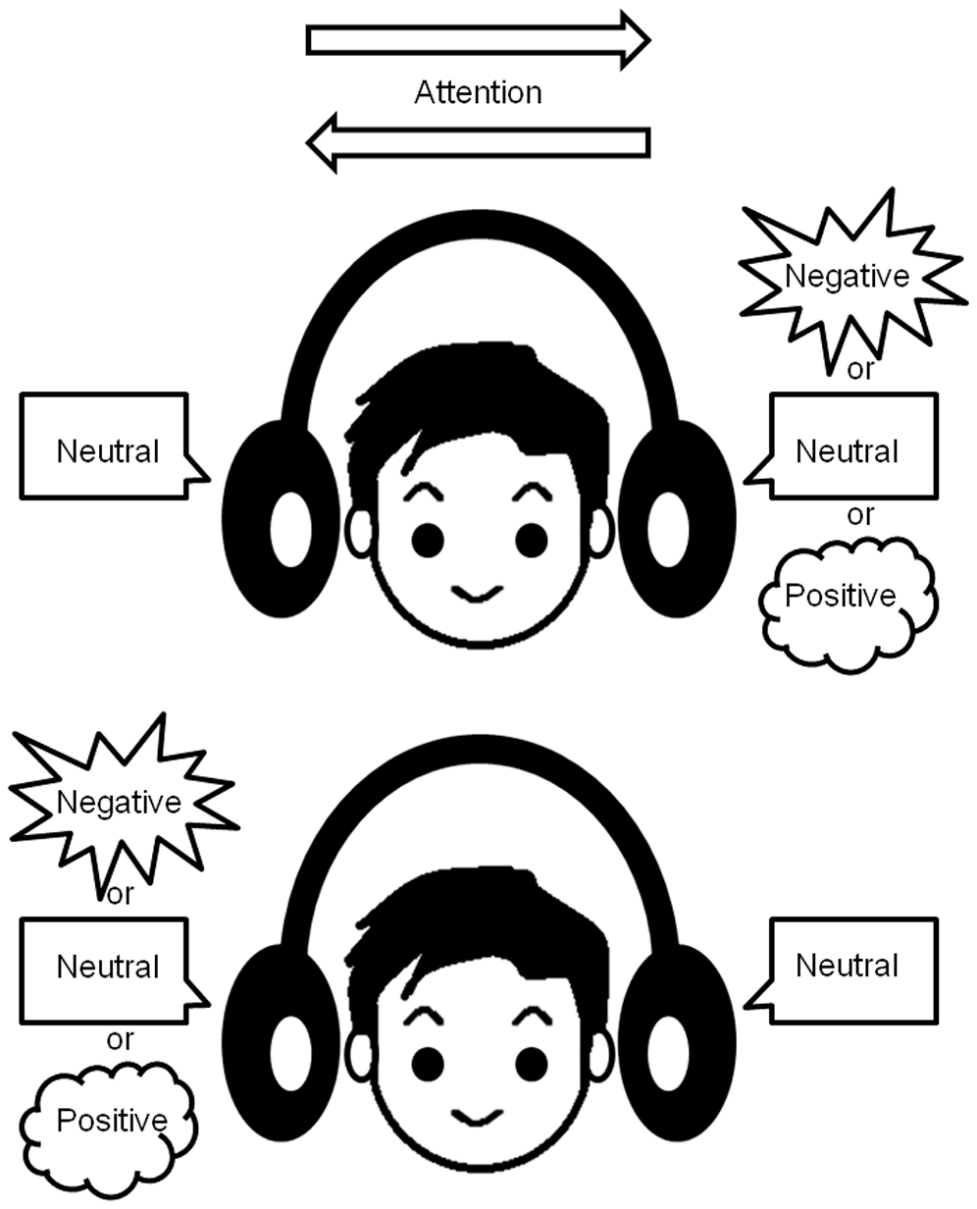
Experimental paradigm. Participants were asked to selectively attend to either the right- or left-sided voice stimulus within a pair. Then, they were required to select the word heard at the attended ear from four presented candidate words on the screen as quickly as possible. Orthogonally to the task demands, voices could have a semantically different emotional valence, either neutral on both sides, negative (or positive) on the attended side and neutral on the other side, or vice versa, neutral on the attended side and negative (or positive) on the other side.

The task design was developed based on that in a previous study (ref. [12]), although that study used emotional prosody in contrast to emotional content of words. Participants responded by pressing a button while listening to a pair of words. To manipulate voluntary attention orthogonally to emotional words, we used a dichotic listening paradigm in which stimuli matched for mean acoustic energy and duration were presented simultaneously to both ears (negative/neutral/positive and neutral or neutral and negative/neutral/positive, on the right and left sides, respectively) in random order (Figure 1). Each stimulus condition (negative-neutral, neutral-negative, positive-neutral, neutral-positive, and neutral-neutral, on the right-left sides, respectively) had 40 trials. The subjects attended at the right ear for half of the 40 trials and the left ear for the other half, for each condition. Subjects were asked to attend at the right or left ear in a pseudo-random order. This information was given by instructions shown on the screen before the presentation of the auditory stimuli. Each word appeared four times in the experimental session and all the trials were presented in random order. Participants were required to selectively attend to either the left or right ear, and select the word heard in the attended ear from four presented words on the screen as quickly as possible. For each event, the time taken to select one word by pressing a button after the auditory stimuli was presented and the number of correct responses were recorded automatically in E-prime 2.0 (http://www.ibsjapan.com/EPRIME.htm) as response time (RT) and correct ratio (CR), respectively.

### Statistical analysis

#### Effects of emotional stimuli, their laterality, and attention in healthy subjects and patients with schizophrenia

First, to verify previous findings that response to negative stimuli was different from the response to neutral or positive stimuli in healthy subjects [12], [14], we examined CR and RT for negative-neutral, positive-neutral, and neutral-neutral word pairs in the healthy participants and the patients with schizophrenia. We performed four repeated measures ANOVAs with two within-subject factors (Stimulus-Type: Negative-Neutral/Neutral-Negative/Positive-Neutral/Neutral-Positive/Neutral-Neutral; Attention-Side: right/left). Two had CR as the dependent variable (one for healthy subjects and one for patients with schizophrenia) and two had RT as the dependent variable (one for healthy subjects and one for patients with schizophrenia). The statistical model was based on that in a previous study (ref. [12]). The threshold for statistical significance was set at p<0.05.

#### Comparisons between subjects with schizophrenia and healthy controls

Analyses for comparisons between patients with schizophrenia and controls were confined to an index, which revealed significant interference of emotional valence in healthy subjects (i.e. RT, see Results). Based on prior findings describing a general slowness in RT in patients with schizophrenia [47], [48], [49], we treated interference indices as dependent variables in the main analysis. Interference indices allow a more specific assessment of the effect of emotional valence on inhibitory processes by minimizing the effect of general slowness. They were calculated by a widely employed formula:
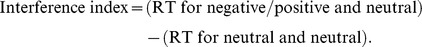
Thus, the condition of neutral and neutral word pairs served as baseline [7], [50].

A repeated measures ANOVA with one between-subject factor (Diagnosis: schizophrenia/control) and two within-subject factors (Stimulus-Type: Negative-Neutral/Neutral-Negative/Positive-Neutral/Neutral-Positive; Attention-Side: right/left) with interference indices as dependent variables was performed. If a significant Diagnosis-by-Stimulus-Type or Diagnosis-by-Stimulus-Type-by-Attention-Side was found, follow-up analyses using repeated measures ANOVAs separately for each Stimulus-Type were performed. The threshold for statistical significance was set at p<0.05.

#### Correlations between interference indices and clinical measurements

Associations between interference indices showing significant group differences and positive symptoms, negative symptoms and general psychopathology on the PANSS, and delusional behavior scores were further tested with Spearman's rank correlation in the patient group. Selective attention to threatening information may lead an individual to form paranoid delusions about the environment [48], [51], [52]. Thus we hypothesized that abnormal interference indices for negative-neutral word pairs would correlate with the severity of positive symptoms and delusional behavior in schizophrenia. Therefore, statistical significance was set at p<0.05 for these correlations. In contrast, because no theoretical hypothesis about the correlations between severity of negative symptoms, general psychopathology, and interference indices exists, statistical significance was set at p<0.025 after Bonferroni correction for two correlations (one condition×two clinical measurements).

Additionally, correlations between interference indices and potential confounding factors, including age, self SES, parental SES, age of onset, duration of illness, and dose of neuroleptics, were tested separately in each group using Spearman's rank correlation. In contrast, because no theoretical hypothesis about the correlations between the other clinical measurements and interference indices exists, the threshold for statistical significance was set at p<0.0056 (Bonferroni correction for nine correlations [six for schizophrenia group {one condition×six clinical measures}; three for control group {one condition×three clinical measures}]).

## Results

### Effects of emotional stimuli, their laterality, and attention in healthy controls and patients with schizophrenia

**Figure 2:**
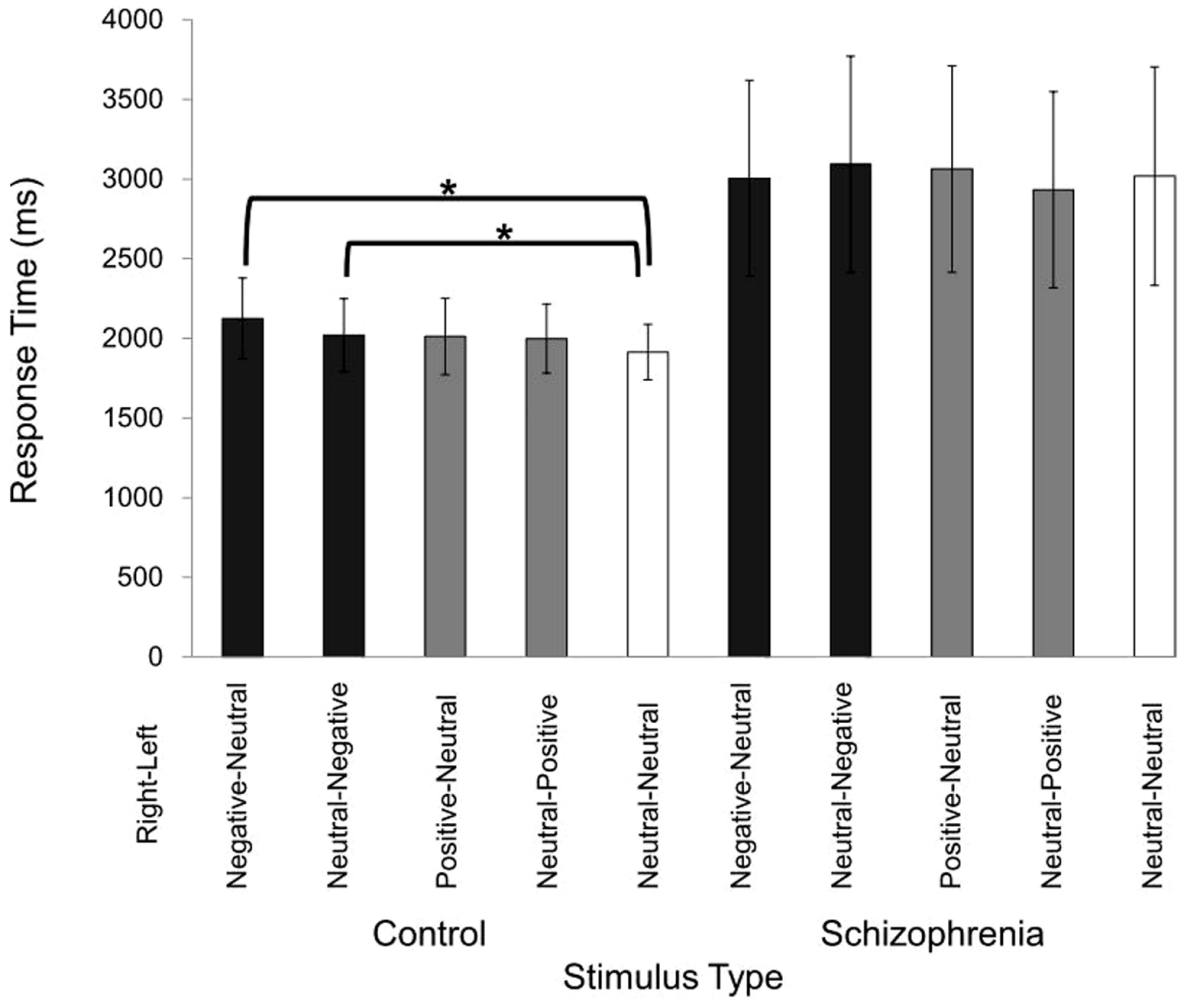
Effect of auditory negative words on response time in healthy subjects and patients with schizophrenia. Response times for Negative-Neutral, Neutral-Negative stimuli on the right-left side were significantly longer than that for Neutral-Neutral word pairs, irrespective of direction of attention in the healthy controls (p = 0.0035, p = 0.025, respectively). In contrast, response time for Positive-Neutral (p = 0.11), Neutral-Positive (p = 0.12) word pairs on the right-left side was not significantly longer than that for Neutral-Neutral word pairs.

For RT, the repeated measures ANOVA showed a significant main effect of Stimulus-Type (F[4,92] = 3.89, p = 0.006) with no significant interaction (F[4,92] = 1.93, p = 0.11). Post-hoc paired t-tests revealed that RT for Negative-Neutral and Neutral-Negative (right-left side) (t[23] = 3.25, p = 0.0035; t[23] = 2.40, p = 0.025, respectively) were significantly longer than that for Neutral-Neutral word pairs, irrespective of direction of attention in healthy controls (Table 2, Figure 2). The same result was obtained if one extreme outlier with the maximum RT for Negative-Neutral stimuli was excluded (main ANOVA, p = 0.03; post hoc paired t-test, p = 0.00048). In contrast, no significant effects were found in schizophrenia group (p>0.16). Repeated measures ANOVAs showed no significant main effects or interactions for CR (p>0.64). A significant interaction between Attention-Side and Stimulus-Type was found in the patient group (F[4,84] = 4.92, p = 0.001). Post-hoc paired t-tests revealed that the CRs for Neutral-Negative (t[21] = 2.51, p = 0.02), Positive-Neutral (t[21] = 2.53, p = 0.02) and Neutral-Positive (t[21] = 2.59, p = 0.017) were higher than CR for Neutral-Neutral word pairs when patients attended to the right ear. The CRs for Negative-Neutral (t[21] = 2.75, p = 0.012) and Neutral-Neutral (t[21] = 3.18, p = 0.005) were higher than CR for Neutral-Negative word pairs when patients attended at left ear.

**Table 2:** Reaction time (msec).

Healthy subjects (n = 24)						Paired t-test (post-hoc)					Patients with schizophrenia (n = 22)			
Stimulus-Type			Attention-Side			Relative to each stimulus-type					Stimulus-Type		Attention-Side	
	Mean	SD		Mean	SD		Neu-Neg	Pos-Neu	Neu-Pos	Neu-Neu	Mean	SD	Mean	SD
Neg-Neu	2125.0	633.9	R	2178.9	607.7	T-value	2.16	1.83	1.89	3.25	3004.4	1471.1	3048.9	1394.7
			L	2071.1	726.9	P-value	0.042	0.08	0.072	0.0035*			2959.8	1588.8
Neu-Neg	2020.2	572.0	R	1988.3	584.5	T-value		0.16	0.48	2.4	3092.2	1624.3	3092.4	1890.9
			L	2052.1	596.2	P-value		0.88	0.63	0.025*			3091.9	1470.7
Pos-Neu	2012.2	600.9	R	2049.0	652.4	T-value			0.33	1.67	3062.8	1551.4	3066.3	1599.2
			L	1975.4	586.7	P-value			0.74	0.11			3059.3	1579.3
Neu-Pos	1998.7	543.1	R	1966.8	647.3	T-value				1.63	2932.6	1473.3	2999.5	1587.8
			L	2030.6	496.3	P-value				0.12			2865.7	1424.4
Neu-Neu	1913.7	435.7	R	1884.7	476.5						3017.8	1640.9	3021.8	1708.0
			L	1942.8	443.6								3013.7	1593.0

ANOVA						
Effect	F	P	Effect Size^a^	F	P	Effect Size^a^
Stimulus-Type	3.89	0.01	0.41	1.71	0.16	0.28
Attention-Side	0.00	0.99	0.00	0.44	0.52	0.14
Stimulus-Type×Attention-Side	1.93	0.11	0.29	0.19	0.94	0.10

Abbreviations: Neg-Neu/Neu-Neg/Pos-Neu/Neu-Pos/Neu-Neu, Negative-Neutral/Neutral-Negative/Positive-Neutral/Neutral-Positive/Neutral-Neutral word pair (right ear-left ear). R/L, Right/Left;

* Statistically significant;

a Cohen defines fs of 0.1, 0.25, 0.4 as small, medium, large, respectively.

### Comparisons between subjects with schizophrenia and controls

**Figure 3:**
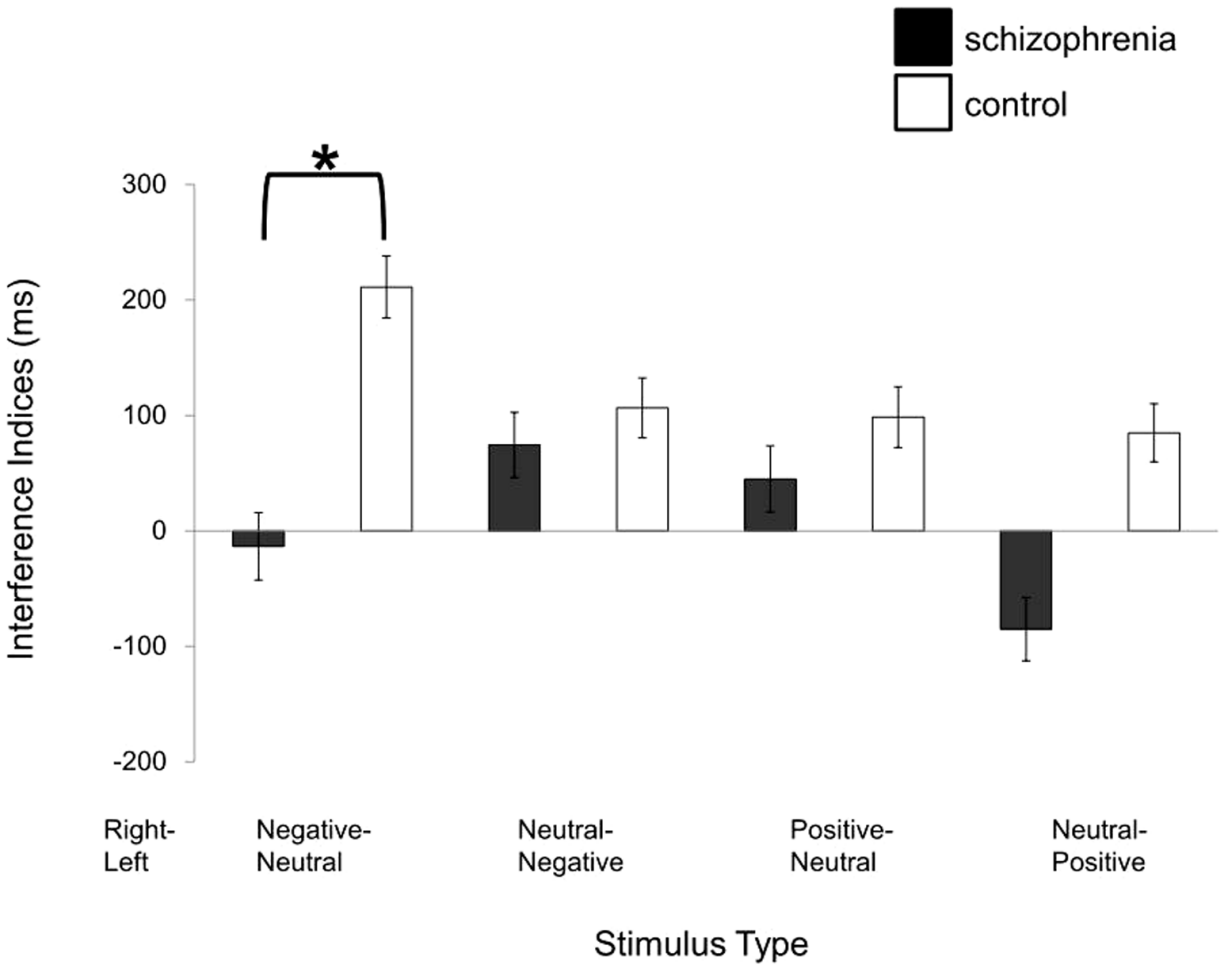
Aberrant interference of auditory negative words in patients with schizophrenia. The interference index in patients with schizophrenia was significantly smaller compared with healthy controls when negative words were presented at the right ear, irrespective of attention side (p = 0.025). Because no main effect of Attention-Side and no interaction between Attention-Side and any other factors were found, the means of the interference indices for attending to the right and left ear are presented.

The repeated measures ANOVA showed a significant main effect of stimulus-type (F[3,132] = 2.69, p = 0.049) and interaction between Diagnosis and Stimulus-Type (F[3,132] = 2.83, p = 0.041), and no other effect and interaction (p>0.35). Post-hoc paired t-tests revealed that interference indices for Negative-Neutral (t[45] = 2.47, p = 0.018), Neutral-Negative (t[45] = 2.30, p = 0.026) and Positive-Neutral (t[45] = 2.14, p = 0.038) (right-left side) were significantly longer than that for Neutral-Positive (right-left side) word pairs, irrespective of direction of attention. For Negative-Neutral (right-left side) stimulus-type, the post-hoc repeated measures ANOVA showed a significant main effect of Diagnosis (F[1,44] = 5.39, p = 0.025). For other stimulus types, there was no significant main effect of Diagnosis or interactions. This analysis demonstrated that the interference index in patients with schizophrenia was significantly smaller than that in controls when negative words were presented to the right ear, irrespective of attention side (Table 3, Figure 3). The statistical conclusions remained the same when the one left-handed subject in the schizophrenia group was excluded and when analysis of covariance including dexterous ear as a covariate was employed. Furthermore, if extreme outliers (i.e. one patients with the minimum interference index and one control with the maximum index for Negative-Neutral stimuli) were excluded, there remains a tendency of the same statistical conclusion (main ANOVA, p = 0.070; post hoc ANOVA, p = 0.066).

**Table 3:** Comparisons of Interference indices between the patients and controls.

Patients with schizophrenia (n = 22)						Controls (n = 24)				Repeated measures ANOVA	
Stimulus-Type			Attention-Side			Stimulus-Type		Attention-Side		(post-hoc)	
	Mean (msec)	SD		Mean (msec)	SD	Mean (msec)	SD	Mean (msec)	SD	Group	
Neg-Neu	−13.4	69.9	R	27.1	650.6	211.3	66.9	294.3	341.5	F	5.39
			L	−53.9	330.6			128.3	416.5	P	0.025*
Neu-Neg	74.4	67.7	R	70.6	492.4	106.5	64.8	103.7	277.0	F	0.12
			L	78.2	694.3			109.3	259.3	P	0.73
Pos-Neu	45.0	68.7	R	44.5	633.9	98.5	65.8	164.4	328.9	F	0.32
			L	45.6	487.7			32.6	309.3	P	0.58
Neu-Pos	−85.2	65.6	R	−22.3	607.7	85.0	62.9	82.2	400.9	F	3.51
			L	−148.0	417.6			87.8	218.1	P	0.068

ANOVA			
Effect	F	P	Effect Size^a^
Stimulus-Type	2.69	0.05	0.25
Attention-Side	0.89	0.35	0.14
Group	2.19	0.15	0.22
Stimulus-Type×Attention-Side	0.47	0.70	0.11
Attention-Side×Group	0.03	0.86	0.03
Stimulus-Type×Attention-Side×Group	0.55	0.65	0.11

Abbreviations: Neg-Neu/Neu-Neg/Pos-Neu/Neu-Pos, Negative-Neutral/Neutral-Negative/Positive-Neutral/Neutral-Positive word pair (right ear-left ear).

R/L, Right/Left;

* Statistically significant;

a Cohen defines fs of 0.1, 0.25, 0.4 as small, medium, large, respectively.

### Correlational analysis

**Figure 4:**
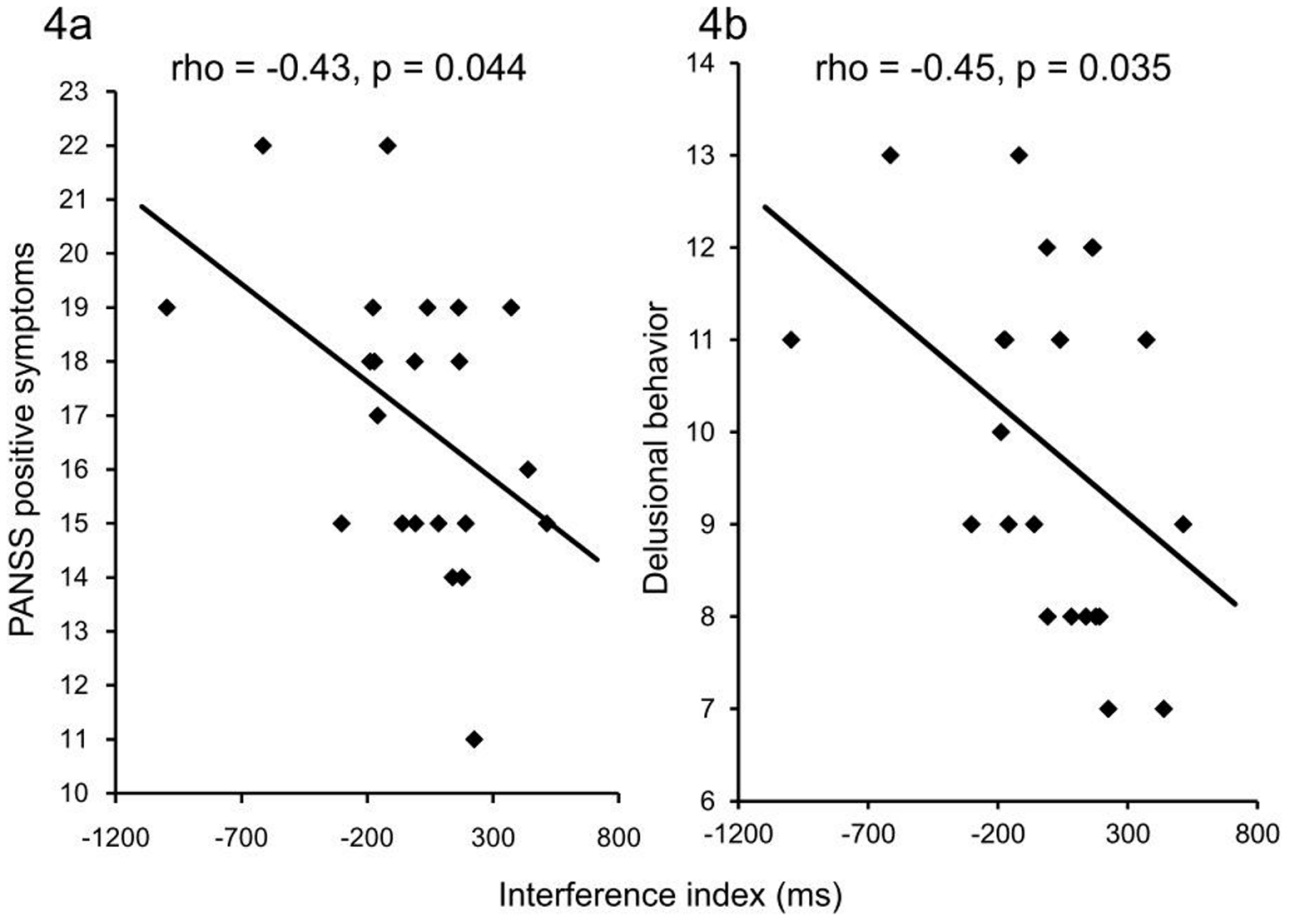
Relationships between the aberrant interference and positive symptoms, delusional behavior. Scatter plots depict correlations between the interference index for negative-right/neutral-left word pairs irrespective of attention side and severity of positive symptoms in the Positive and Negative Syndrome Scale (PANSS) (rho = −0.43, p = 0.044) (a) and severity of delusional behavior (rho = −0.45, p = 0.035) (b) in the patients with schizophrenia.

Because no main effect of Attention-Side and no interaction between it and any other factors were found, means of the interference indices for participants' attending on right- and left-ear were used. The interference index for negative-neutral pairs when negative words were presented to the right ear irrespective of attention side significantly negatively correlated with the PANSS positive symptoms severity (rho = −0.43, p = 0.044) and delusional behavior (rho = −0.45, p = 0.035) (Figure 4), but was not correlated with PANSS negative symptoms (p = 0.39>0.05/2) or general psychopathology (p = 0.14>0.05/2). There was no significant correlation between the interference index and potential confounds in each group (p≥0.10>0.05/9).

## Discussion

To our knowledge, the present study is the first to demonstrate that response time was longer when emotionally negative but not positive words were auditorily presented than when only neutral ones were presented. Furthermore, for the first time, a significantly smaller interference effect of negative emotional words on auditory attention was demonstrated in patients with schizophrenia compared with healthy controls, while interference of positive emotional words was not significantly different between patients and controls. Furthermore, reduced interference of negative auditory words significantly correlated with severe positive symptoms and delusional behavior in patients.

We demonstrated that auditorily presented negative words were semantically processed whether they were or were not the focus of attention in healthy subjects. This interference effect of implicitly processed negative emotional stimuli has been observed, similar to visually presented negative words [15], [53]. The current results are consistent with previous findings that detection of potential threats, such as angry faces [14] or angry prosody [12], occurs even when they are not initially the focus of attention.

With respect to the laterality effect for the attended ear, our findings are partially inconsistent with those of previous dichotic listening studies [12], [13], [46], [54], [55]. For instance, in a paper by Hugdahl et al., the authors used simple consonant–vowel syllables and found that subjects tended to answer the sound from the right ear more often than that from left ear when subjects were not forced attention to either of ear [55]. However, the current study forced subjects to attend right or left ear. The previous studies that also forced subjects to attend right or left ear similarly failed to find laterality effect on CR [12], [13], [46]. Although one previous study [54] reported laterality effect on CR utilizing the similar experimental design as these studies [12], [13], [46] in participants predominantly consists of males. The current study as well as the previous studies [12], [13], [46] included a number of female subjects. It has been suggested that the emotional process is less lateralized in female than male (reviewed in [56]). Unlike previous studies [12], [13], [46], [54], we also did not find significant laterality effects on the RT. Several differences between the current and previous dichotic listening tasks might explain this discrepancy in the findings. First, our study investigated the effect of semantic contents of emotional words on auditory attention, while these previous studies investigated that of emotional prosody [12], [13], [46], [54]. The processes underlying the semantic aspects of verbal stimuli may not be affected by attention-side so much as by the processing of prosody. Second, in the current study, we required the subjects to select target words from four words shown on a screen, while in previous studies, the subjects were simply asked to judge the gender of a voice by pressing a button [12], [13], [46], [54]. Thus, the current study was more demanding in terms of reading speed and working memory than were these previous studies. IQ and years of education did not differ between the patient and control groups. Furthermore, we employed interference indices, which treat RTs for neutral-neutral stimuli as a baseline. This allowed us to minimize the individual differences in these factors that were unrelated to emotional valence [7], [50], although the possibility that these factors confounded the laterality effect of attention-side cannot be completely ruled out. We observed small to moderate effect sizes for interaction between Attention-Side and Stimulus-Type on RT in the healthy subjects, effect of Attention-Side on RT in the patients with schizophrenia and interference indices across groups (Table 2 and 3). It is also possible that we did not find laterality effects due to insufficient statistical power for these indices.

In patients with schizophrenia, this interference effect of auditory negative words was significantly smaller than in healthy individuals. Most previous studies using an emotional Stroop task and investigating attention-emotion interaction by visually presenting emotional words have shown increased interference of negative words in patients with schizophrenia compared with healthy controls [22], [57]. Another study demonstrated that patients with schizophrenia were unable to select nonsalient over salient visually presented stimuli [58]. The apparent discrepancy between the current and previous studies might derive from the difference in the modality used in the task, with emotional words presented auditorily in the current study. Indeed, significantly less interference of negative emotional stimuli in other modalities such as face, prosody, or odour have been shown in patients with schizophrenia compared with healthy controls [9], [59], [60]. A previous study reported prolonged RTs for emotional prosody in patients with schizophrenia compared with healthy controls. However, this study did not employ interference indices, despite the observation of prolonged RTs (which were observed even for neutral stimuli) in patients with schizophrenia [54]. Another possible explanation for the discrepancy is that less interference in patients with schizophrenia was attributable to general deficits in emotional recognition [61], [62], although the reduced interference was not observed to positive words in either ear or negative words in left ear.

The smaller interference effect when negative words were presented to the right ear might reflect functional and structural left-lateralized abnormalities in brain regions related to language processing, including the inferior frontal gyrus and superior temporal gyrus in patients with schizophrenia (reviewed in [63]). Although it has been suggested that emotional semantic stimuli are predominantly processed in right prefrontal areas [64], [65], semantic processing itself is thought to be dominant in the left hemisphere [66]. In particular, left lateralized abnormalities have been shown in brain activities elicited by semantic contents of auditorily presented word stimuli [67], [68], [69], [70]. Deficits in these brain regions have been associated with the formation of psychotic symptoms, especially delusions, through abnormal semantic processing [19], [20], [21], [29], [31]. Although speculative, the current correlation between less interference of negative words and severe psychotic symptoms and delusional behavior infers that less interference of negative words when they were presented to the right ear resulted from deficits in the left language-related brain regions in patients with schizophrenia. However, neuroimaging studies are needed to examine the neural substrates related to the smaller interference effect of auditory negative words in patients with schizophrenia. Our finding that reduced interference correlated with severe positive symptoms in patients with schizophrenia might be related to the altered sensorimotor gating [71] for negative word stimuli. Sensorimotor gating is defined as the pre-attentive ability of the brain to modulate its sensitivity to an incoming stimulus, and is hypothesized to be a protective mechanism that prevents sensory overload of higher brain functions by filtering out the irrelevant sensory input [71]. Deficits in sensorimotor gating could lead to perceptual and attentional impairments associated with schizophrenia [71], [72] and the associated positive symptoms [73].

We must address the methodological limitations of our study. First, the sample size was relatively small. Results should be replicated in future studies with larger sample sizes, although previous studies investigating interaction between visual emotional words and attention in similar sample sizes [4], [7], [47]. Second, participants included patients with chronic schizophrenia taking antipsychotic medications. Interference indices did not show any significant correlation with dose of neuroleptics or duration of illness in the study participants. However, effects of chronic illness (reviewed in [74]) and antipsychotic medications (reviewed in [75]) on the present findings cannot be totally ruled out. Future studies should employ patients with first episode schizophrenia to minimize effects of chronicity and medications.

In summary, the present study showed that the interference effect of negative words on auditory attention was abnormally smaller in patients with schizophrenia compared with healthy subjects, and was correlated with severe positive symptoms and delusional behavior. The current findings indicate that abnormality in the interaction between semantic-based emotional processing and auditory attention plays a role in the pathophysiology of psychotic symptoms such as delusions in schizophrenia.

## Supporting Information

Appendix S1 **Four question items used to decide the dextrous ear.** These items were based on those in previous literature (Brysbaert, 1994; Coren, 1993; Polemikos and Palaeliou, 2000).
(DOCX)

Appendix S2 **Words list in original English from the Affective Norms for English Words (ANEW; Bradley and Lang, 1999).** (In alphabetical order).
(DOCX)

Appendix S3 **Japanese words list to show the Japanese four morae.** (In alphabetical order).
(DOCX)
