# Supplementary material for: Aberrant Interference of Auditory Negative Words on Attention in Patients with Schizophrenia
Source: PLoS One. 2013 Dec 23;8(12):e83201. doi: 10.1371/journal.pone.0083201 (PMC3871545; doi:10.1371/journal.pone.0083201)
Supplement: Appendix S1 — Four question items used to decide the dextrous ear. These items were based on those in previous literature (Brysbaert, 1994; Coren, 1993; Polemikos and Palaeliou, 2000). (DOCX) [file pone.0083201.s001.docx]

**Appendix S1.** Four question items used to decide the dextrous ear.

“Place against a closed door to listen in on a conversation.”

“Place into the ear phone of a transistor radio.”

“Place against a chest to hear a heartbeat.”

“Press against a box to find out if a clock in the box in ticking.”
