# Supplementary material for: Aberrant Interference of Auditory Negative Words on Attention in Patients with Schizophrenia
Source: PLoS One. 2013 Dec 23;8(12):e83201. doi: 10.1371/journal.pone.0083201 (PMC3871545; doi:10.1371/journal.pone.0083201)
Supplement: Appendix S2 — Words list in original English from the Affective Norms for English Words (ANEW; Bradley and Lang, 1999). (In alphabetical order). (DOCX) [file pone.0083201.s002.docx]

**Appendix S2.** Words list in original English from the Affective Norms for English Words (ANEW; Bradley and Lang, 1999). (In alphabetical order)

| **Negative words** | **Positive words** | **Neutral words** | |
| --- | --- | --- | --- |
| Angry  Coffin  Fat  Garbage  Germs  Hardship  Hate  Hostile  Hurt  Impotent  Insult  Jail  Lie  Loser  Misery  Scorn  Scum  Thief  Upset  waste | Achievement  Birthday  Bless  Couple  Easy  Good  Humor  Life  Money  Passion  Peace  Safe  Sex  Soft  Star  Sun  Sunlight  Triumph  Truth  wish | Arm  Basket  Bathroom  Board  Book  Building  Cellar  Chaos  Cliff  Concentrate  Contents  Dark  Detail  Engine  Fabric  Finger  Hairpin  Hammer  Haphazard  Highway  Hydrant  Industry  Item  Journal  Jug  Kettle  Knot  Lightbulb  Lightning  Lump | Machine  Manner  Market  Medicine  Metal  Method  Milk  Month  Name  Obsession  Owl  Pig  Privacy  Razor  Revolt  Revolver  Runner  Shadow  Spray  Tamper  Tank  Teacher  Theory  Truck  Trunk  Unit  Vampire  Wagon  Wine  Writer |

Although some words were adjectives in English, we adjusted these to noun in Japanese when these were utilized in the task.
