# Supplementary material for: Aberrant Interference of Auditory Negative Words on Attention in Patients with Schizophrenia
Source: PLoS One. 2013 Dec 23;8(12):e83201. doi: 10.1371/journal.pone.0083201 (PMC3871545; doi:10.1371/journal.pone.0083201)
Supplement: Appendix S3 — Japanese words list to show the Japanese four morae. (In alphabetical order). (DOCX) [file pone.0083201.s003.docx]

**Appendix S3.** Japanese words list to show the Japanese four morae. (In alphabetical order)

| **Negative words** | **Positive words** | **Neutral words** | |
| --- | --- | --- | --- |
| Ba-i-ki-n  Ge-n-kyo-u  Go-mi-ta-me  Go-u-to-u  Hu-n-ga-i  Ka-n-ki-n  Ka-n-o-ke  Ke-i-be-tsu  Ko-n-na-n  Ko-n-ra-n  Ma-ke-i-nu  Na-ma-go-mi  Na-ma-i-ki  No-u-na-si  So-n-ga-i  Su-i-jya-ku  Te-ki-ta-i  U-so-tsu-ki  Za-n-ne-n  Ze-i-ni-ku | A-n-si-n  A-n-ze-n  He-i-o-n  Hyo-u-ki-n  I-ki-ga-i  Jyo-u-ne-tsu  Ka-i-ra-ku  Ka-ne-mo-chi  Ka-n-da-i  Ko-i-bi-to  Ni-k-ko-u  O-te-ga-ra  Se-i-ji-tsu  Se-i-ko-u  Syu-ku-hu-ku  Ta-i-yo-u  Ta-n-jyo-u  Yu-u-bo-u  Yu-u-me-i  Ze-n-ryo-u | Bu-do-u-syu  Bu-ta-ni-ku  Da-i-ho-n  Da-i-me-i  De-n-kyu-u  Do-u-ta-i  E-i-zo-u  E-n-ji-n  Ga-ku-se-tsu  Gu-u-ze-n  Gyu-u-nyu-u  Ha-n-ma-a  He-a-pi-n  Hi-ge-so-ri  Hi-ji-ka-ke  Hi-to-tsu-ki  Ho-u-ho-u  Hu-ku-ro-u  I-na-zu-ma  I-wa-ka-be  Ji-do-u-sya  Ka-i-do-u  Ka-i-te-n  Ka-ku-me-i  Ka-ta-ma-ri  Ki-n-zo-ku  Ko-ku-ba-n  Ko-n-to-n  Ko-u-mo-ku  Ku-ra-ga-ri | Kyo-u-i-n  Mi-zu-sa-shi  Mo-no-ka-ki  Mu-su-bi-me  Na-i-mi-tsu  Na-i-yo-u  No-u-syu-ku  O-ri-mo-no  O-to-si-do  O-ya-yubi  Ra-n-na-a  Sa-n-gyo-u  Se-ri-i-ti  Si-n-bu-n  Su-i-do-u  Su-i-so-u  Su-pu-re-e  Syo-u-sa-i  Syu-u-tya-ku  Ta-i-se-i  Ta-n-pa-a  Ta-te-mo-no  To-ri-hi-ki  Tsu-ri-ka-go  Tyo-zo-u-ko  Ya-ku-za-i  Ya-ri-ka-ta  Yo-ku-si-tsu  Yu-ni-t-to  Yu-wa-ka-si |
